# Experimental Investigation on the Use of a PEI Foam as Core Material for the In-Situ Production of Thermoplastic Sandwich Structures Using Laser-Based Thermoplastic Automated Fiber Placement

**DOI:** 10.3390/ma15207141

**Published:** 2022-10-13

**Authors:** Berend Denkena, Carsten Schmidt, Christopher Schmitt, Maximilian Kaczemirzk

**Affiliations:** 1 Institute of Production Engineering and Machine Tools, Leibniz University Hannover, An der Universität 2, 30823 Garbsen, Germany; 2 Institute of Production Engineering and Machine Tools, Leibniz University Hannover, Ottenbecker Damm 12, 21684 Stade, Germany

**Keywords:** thermoplastic automated fiber placement, thermoplastic sandwich composites, PEI foam, additive manufacturing, VCSEL, in-situ

## Abstract

Laser-based thermoplastic automated fiber placement (TAFP) is nowadays mainly used to produce pure carbon fiber-reinforced plastic (CFRP) structures. This paper investigates the feasibility of a novel application: The deposition of thermoplastic prepreg tapes onto a thermoplastic foam for the production of thermoplastic sandwich structures. Therefore, simple deposition experiments of thermoplastic PEEK/CF prepreg tapes on a PEI closed-cell foam were carried out. 3D surface profile measurements and peel tests according to DIN EN 28510-1 standard were used to investigate the joining area and bonding quality. The results show that a cohesive bond is formed between the deposited tapes and the foam core, however the foam structure in the area of the deposited tapes deforms in dependence of the process parameters, and increasingly with higher deposition temperatures. Due to the deformations that occur during tape deposition, the thermomechanical foam behavior under the TAFP process conditions was investigated in more detail in a subsequent study for an extensive parameter space using a simple experimental setup. Results show that for suitable process parameters, namely a short contact time and a high temperature, the foam deformation can be minimized with the simultaneous formation of a thin melting layer required for cohesive bonding. The inner foam core structure remains unaffected.

## 1. Introduction

**Figure 1:**
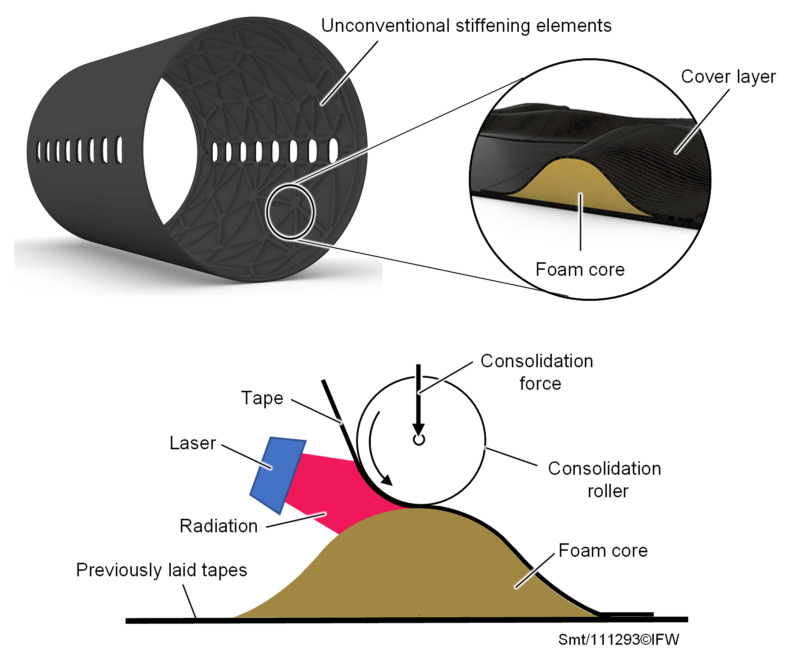
Unconventional stiffening elements of an aircraft panel and TAFP manufacturing methodology.

Components and structures made of carbon fiber-reinforced plastic (CFRP) offer enormous potential for lightweight construction due to their high specific strength. In recent years, the trend has shifted from thermoset to thermoplastic materials in order to be able to use the advantage of recyclability and the joining of two components through local melting of the matrix [1,2]. A particularly good ratio of mechanical properties to weight is achieved by sandwich structures, which consist of two outer fiber-reinforced cover layers and an inner, thicker foam core. The inner core increases the distance between the two cover layers and thereby increases the area moment of inertia and the flexural rigidity of the structure, while the weight is only increased minimally [3]. Furthermore, the core determines the shear strength and the pressure behavior. The cover layers, on the other hand, carry the bending loads. In addition, thermoplastic sandwich structures are characterized by high damage tolerance, thermal insulation and acoustic damping [4,5]. However, the production of thermoplastic sandwich structures is largely limited to planar structures, which can be produced continuously and automatically by using double-band laminating or compression molding [6,7]. With regard to the production of geometrically complex and individual sandwich structures [8] (Figure 1), however, these processes show economic and technological limits, which are reflected in particular in highly complex molds and a large number of interlinked process steps [9,10,11].

In contrast, automated fiber placement (AFP) shows great potential for the production of geometrically complex and individual CFRP structures [12]. Pre-impregnated tapes (prepreg) are laid down along the load path, so that shape and load-specific CFRP structures are created in an additive process. Using a laser heating source, thermoplastic prepreg tapes can be processed with the thermoplastic automated fiber placement (TAFP) in-situ and without a time-consuming autoclave process. The application range of the laser-based TAFP is currently focused on the manufacture of CFRP structures. The fabrication of hybrid structures is presented, for example, in [13,14] as a metal-CFRP hybrid. The deposition of thermoplastic prepregs on thermoplastic foam has not yet been considered. Although such a process is very attractive in terms of flexibility and productivity, it also presents some challenges: one of them is the formation of a cohesive bond between the foam core and the cover layer.

The quality of the cohesive bond is mainly determined by the temperature prevailing at the contact point; however, the surface condition, consolidation pressure and consolidation time also have an influence [15]. For the production of CFRP structures by means of a modern NIR laser-based TAFP, the supplied tape and the already deposited substrate are heated by a laser source. Since the thermoplastic matrix transmits a large portion of the NIR laser radiation (wavelength ≈ 980 nm) [16], the heating of the tapes occurs via heating of the absorbing carbon fibers and subsequent heat conduction into the surrounding matrix [16,17]. Accordingly, there is no surface heat input, but a volumetric one depending on the fiber matrix distribution, the surface properties and the angle of incident [16,17,18,19]. The prepregs and the foam substrate differ greatly in these properties, especially due to the absence of carbon fibers in the foam structure. Therefore, different temperature distributions on foam and tape are expected for the same machine and process settings. Accordingly, for a good cohesive bond, a specific and independent adjustment of the heating parameters for the tape and the foam is necessary. Conventional laser systems are not suitable for this purpose due to their homogeneous radiation characteristics [16], but novel Vertical-Cavity Surface-Emitting Laser (VCSEL) systems show the possibility to realize a zone-individual temperature control [20,21].

Another major challenge arises from the transition from heating to consolidation. The consolidation roller used to apply the consolidation pressure causes a shadow area due to its geometry [16,18,22]. Within this range, the laser radiation no longer heats the tape and substrate directly, so that a drop in temperature occurs before the consolidation zone.

Since a high cohesive bond is determined by the intermolecular diffusion of polymer chains between the joining partners, heating is applied above the melting temperature before the shadow area, so that the temperature in the consolidation zone is also still at melting temperature [23]. In addition to the risk of matrix degradation [24], the high temperatures and the imposed consolidation pressure also result in the risk of the foam core colliding and geometric deviations in the contour of the structure to be produced.

This research paper demonstrates the deposition of thermoplastic prepregs on a similar foam core and the formation of cohesive bonding in this process. It uses an isolated study to investigate the geometric change of the foam core under the specific thermomechanical loads from the TAFP. This will contribute to the implementation of thermoplastic hybrid structures in the TAFP.

## 2. Materials and Methods

### 2.1. Foam and Prepreg Tapes

The foam and prepreg tape used in this study were selected with respect to the high requirements for materials processed in aerospace applications. The thermoplastic polyetherimide (PEI) closed-cell foam R82.110 from AIREX AG (Sins, Switzerland) was used for the investigations carried out in this work. In addition, a polyetheretherketone/carbon fiber (PEEK/CF) ¼″ unidirectional prepreg tape (Tenax^®^ TPUD PEEK/IMS65 (Teijin Carbon Europe GmbH, Wuppertal, Germany)) was used to investigate the formation of a cohesive bond between the tape deposited on the foam core using the TAFP. Since miscibility is given for the two polymer systems of the foam (PEI) and prepreg tape (PEEK), the requirement for the formation of a cohesive bond between the materials to be joined is fulfilled [25].

### 2.2. Fiber Placement Analysis

In order to investigate the feasibility of depositing thermoplastic prepreg tapes onto a thermoplastic foam core by means of the TAFP for the production of thermoplastic sandwich structures, simple placement trials are carried out. The aim here is to achieve cohesive bonding between the two joining partners and to identify the effects occurring during this process.

#### 2.2.1. Laser-Based TAFP System

**Figure 2:**
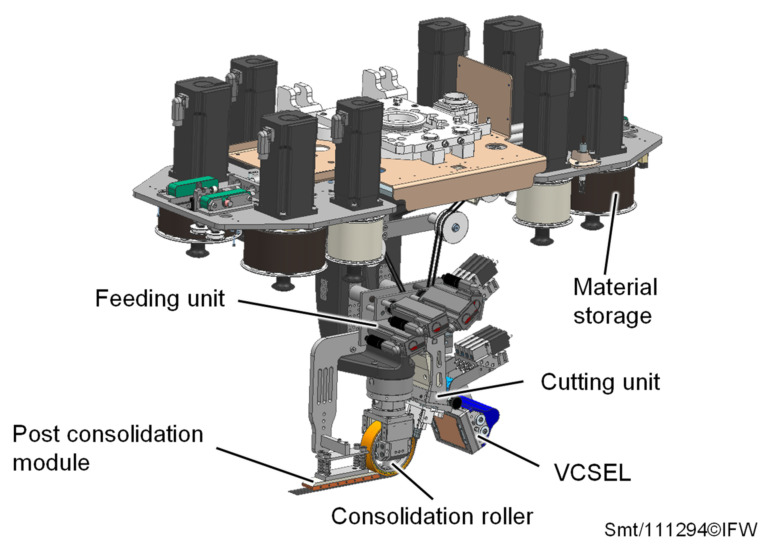
TAFP laying head developed at the Institute of Production Engineering and Machine Tools.

A TAFP laying head developed at the Institute of Production Engineering and Machine Tools is used to deposit the thermoplastic prepreg tape onto a thermoplastic foam core (Figure 2).

The modular laying head consists of six independent modules: material storage, feeding unit, cutting unit, heating system, consolidation roller and post consolidation module. The material storage and the feeding unit are actively driven, and convey up to four ¼″ prepreg tapes through the cutting unit to the heating and consolidation zone. A 2.4 kW Vertical-Cavity Surface-Emitting Laser (VCSEL) from the company TRUMPF (Ditzingen, Germany) is used to heat the deposited tapes. By controlling twelve vertically arranged diode field zones, the prepreg tapes and the substrate can be individually tempered in the heating zone [21]. The consolidation roller (outer diameter = 110 mm, width = 30 mm) is coated with a 3 mm thick rubber and is equipped with a piezo-electric actuator. This allows not only static pressure to be applied in the process, but also vibrations to be introduced perpendicularly to the deposited tapes, with a frequency of up to 2 kHz and an amplitude of 40 µm to improve the quality of the deposited laminate [26]. Additionally, the laying head is equipped with a heatable post consolidation module in order to be able to specifically temper the laminate during the cooling phase. However, the last two modules mentioned are not of further interest in the following.

In the experimental study, the laying head is used to deposit single ¼″ PEEK/CF unidirectional prepreg tapes onto a flat AIREX R82.110 foam sheet (20.2 × 300 × 100 mm) under the process parameters shown in Table 1.

The temperature on the tape T_T_ right in front of the nip point is varied. Two temperatures are set to be between the glass transition temperature of the foam material (T_g,PEI_ = 217 °C) and the melting temperature of the prepreg tape matrix (T_m,PEEK_ = 343 °C). One temperature is set to be slightly above the melting temperature of PEEK to meet the requirements for cohesive bonding [25]. The correlation between the specified tape temperature T_T_ and the laser power to be set is determined in preliminary trials. Temperature profiles are therefore recorded with a FLIR A35 infrared camera (Teledyne FLIR LLC, Wilsonville, Oregon, U.S.) during deposition. Except for the tape temperature T_T_ in front of the nip point, all other parameters remain unchanged during the placement investigations. The VCSEL is oriented in such a way that mainly the supplied PEEK/CF prepreg tape is irradiated. The foam material, on the other hand, is only irradiated in a very small area close to the nip point. This is to prevent temperatures above the glass transition temperature of the foam material in front of the joining zone, and to prevent an associated potential foam collapse.

#### 2.2.2. Foam and Tape Deformation Characterization

**Figure 3:**
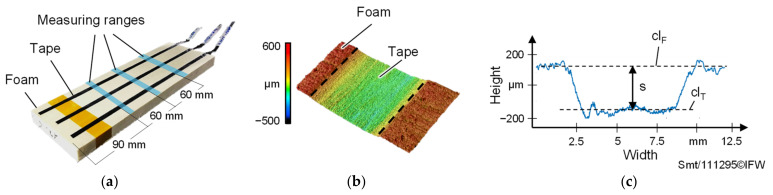
(**a**) Foam sheet with prepreg tapes deposited and position of measuring ranges to create the surface profiles; (**b**) Surface profile of a prepreg tape deposited on the foam; (**c**) Averaged height profile to determine the distance s between tape and foam surface.

In order to determine the deformation that occurs when the heated prepreg tapes are deposited on the foam, non-contact 3D surface profile measurements are carried out at three different measuring ranges for each deposited tape (Figure 3a) using the VK-X1000 3D laser scanning microscope from Keyence (Keyence Cooperation, Osaka, Japan).

For each surface profile (Figure 3b), five height profiles are recorded at intervals of 1 mm and an average height profile (Figure 3c) is determined. Subsequently, the distance s between the horizontal foam and tape surface is calculated using compensation lines cl_F_ and cl_T_ within the Keyence Multi-File-Analyzer^®^ software (Version number: 2.2.0.93, Keyence Cooperation, Osaka, Japan). Finally, an average distance s_m_ consisting of three distance s values is calculated according to Equation (1) for each deposited tape. The standard deviation σ_s,m_ is used for error estimation.

(1) $$s_{m}=\frac{1}{3}\sum_{i=1}^{3}s_{i}$$

#### 2.2.3. Bond Strength Characterization

**Figure 4:**
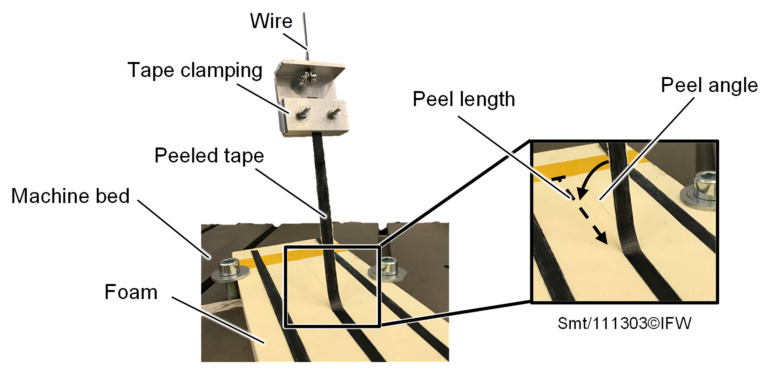
Test setup to peel off tapes from the foam.

Based on DIN EN 28510-1 standard, peel tests are carried out on a universal testing machine (Instron 8870 (Norwood, MA, USA)) with appropriate force and displacement measurement equipment to evaluate the bonding quality of the deposited prepreg tapes to the foam. For this purpose, the tapes are peeled off the foam (Figure 4) at a starting peel angle of 90° at a test speed of 50 mm/min.

To minimize the angular change between the tape to be peeled off and the foam fixed on the testing machine bed, a 600 mm long wire is inserted between the tape clamping and the clamping of the testing machine. This results in a peel angle of 90° at the beginning of the test and a peel angle of 83.24° for a tape peel length of 85 mm at the end of the test. To determine the bonding quality, an average peel force F_p_ is then determined within the peel length range between 30 and 60 mm, in order to neglect possible boundary effects that may arise during the start of the placement process. Subsequently, a specific peel force F_p,spe_ is calculated by relating the averaged peel force to the tape width. The standard deviation σ_F,p,spe_ is used for error estimation.

### 2.3. Foam Compression Analysis

Based on the results of the fiber placement investigations and the foam deformations occurring during deposition, an additional isolated experimental study is conducted to investigate the thermomechanical foam behaviour under process parameters prevailing in the TAFP.

#### 2.3.1. Experimental Setup

**Figure 5:**
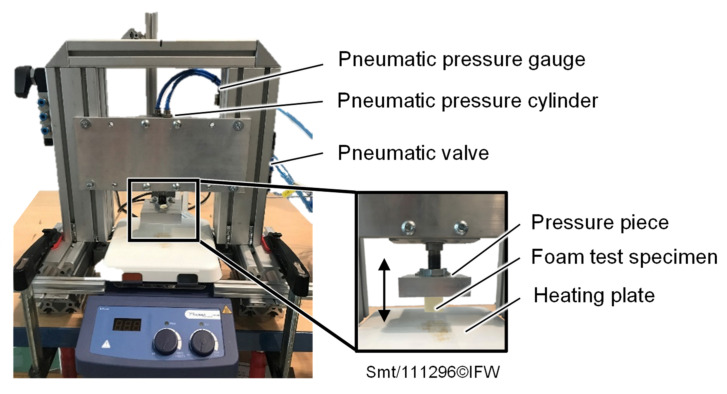
Test setup for the experimental investigation of thermomechanical foam behavior under TAFP process parameters.

A simplified test setup (Figure 5) is used to experimentally investigate the thermomechanical effects of depositing a heated prepreg tape onto a foam core. A possible preheating of the foam core in the process by the VCSEL is neglected and a sole heating of the supplied prepreg tape is assumed. The experimental setup consists of a frame structure with a pneumatic pressure cylinder (FESTO DZF-50-40-A-P-A (Festo GmbH & Co. KG, Esslingen, Germany)). The foam test specimen is attached to the cylinder and then pressed onto the heating plate (Phoenix RSM-04H (Phoenix Instrument GmbH, Garbsen, Germany)). The test stand is equipped with a laser displacement sensor (Micro-Epsilon ILD 1320-200 (Micro-Epsilon GmbH & Co. KG, Ortenburg, Germany)) and a pressure sensor (FESTO SDE5-D10-NF-Q6E-V-K (Festo GmbH & Co. KG, Esslingen, Germany)) to record the displacement of the cylinder and to check the pressure set manually with a pressure gauge.

To perform the experimental study, foam test specimens in a cuboid shape with a pressure area of 20 mm × 20 mm and an initial height h_i_ of 20.2 mm are pressed onto the hot plate under variation of the parameters shown in Table 2. Three foam test specimens are tested for each parameter set. The selected pressure and contact time ranges are based on typical pressures and contact times applied in TAFP [24,27,28]. The defined temperature range fits both the use of the PEI foam when depositing prepreg tapes of the same matrix system (PEI), and different but miscible matrix systems (PEEK).

To prevent the molten foam test specimens from sticking to the heating plate at high temperatures, the surface of the heating plate is coated with a high temperature-resistant release agent before each test.

#### 2.3.2. Foam Deformation and Melting Layer Characterization

**Figure 6:**
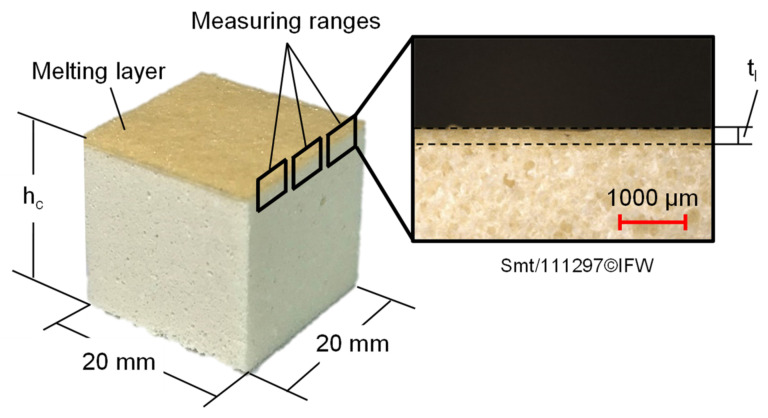
Foam test specimen in compressed state with formed melting layer and position of measuring ranges to determine the thickness of the melting layer by means of lateral optical microscope images.

To investigate the influence of the TAFP parameters on the change of the foam geometry, the initial height h_i_ and the height h_c_ of the compressed foam specimens (Figure 6) are measured with a Holex ABS calliper (Hoffmann SE, Munich, Germany) with a resolution of 0.01 mm. Since the test specimens are measured just before and after the compression test, only the plastic foam deformation is recorded, and therefore the elastic foam deformation is not considered in this study. In addition, three lateral optical microscope images (Keyence VK-X1000 with 2.5× magnification) of each of the compressed foam specimens at regular distances across the specimen width are taken to determine the thickness of the forming melting layer t_l_. First, the transition between the melting layer and the undeformed foam structure is identified (dotted line in Figure 6). Subsequently, the distance between the identified transition within the foam test specimen and the foam test specimen surface (corresponding to the thickness of the melting layer t_l_) is determined by using parallel compensation lines within the Keyence Multi-File-Analyzer^®^ software. Finally, an average melting layer thickness t_m_ is calculated for each parameter set. For error estimation of the determined foam deformation and melting layer thickness, the standard deviation is used in each case.

## 3. Results

### 3.1. Fiber Placement Analysis

#### 3.1.1. Effects of Increasing Temperature on Geometrical Deformations

**Figure 7:**
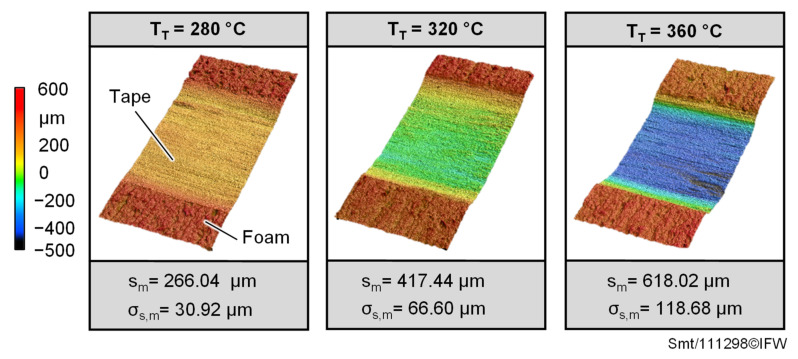
Exemplary surface profiles of the deposited prepreg tape and the underlying foam structure for different placement temperatures and associated distances s_m_ between foam and tape surface with corresponding standard deviations σ_s,m_.

Placement investigations of tapes on the foam core were successful for all varied temperatures, since the tapes stick to the foam. However, the temperature of the Tape T_T_ in the contact area has a major effect on foam stability and the resulting change in geometry (Figure 7). While there is still no great collapse of the foam structure and the associated change in geometry is visible at a temperature of 280 °C, this effect is already much more pronounced at a temperature of 320 °C.

The largest deformations are observed when the tape is deposited on the foam at a temperature above the melting temperature of the PEEK/CF prepreg tape namely 360 °C. In this case, the foam structure collapses on the surface under the influence of the high contact temperature and consolidation pressure, causing the prepreg tape to be strongly pressed into the foam structure. Furthermore, it can be seen in the surface profiles of all the tests carried out that the foam structure does not fail outside the joining zone, although the consolidation pressure is applied there as well, given that the consolidation roller is wider than the prepreg tape to be deposited. The reason therefore is that during the placement process mainly the prepreg tape was heated up, and the foam structure only experiences high temperatures in the contact area of the heated tape. Preheating of the foam structure in the process by the VCSEL does not take place. The average distances s_m_ confirm the increase in the deformation with increasing temperature over the deposition length. However, the associated large standard deviations also show that the deformations vary. This is a direct result of slight consolidation pressure and temperature changes during deposition.

#### 3.1.2. Effects of Increasing Temperature on Bonding Strength

**Figure 8:**
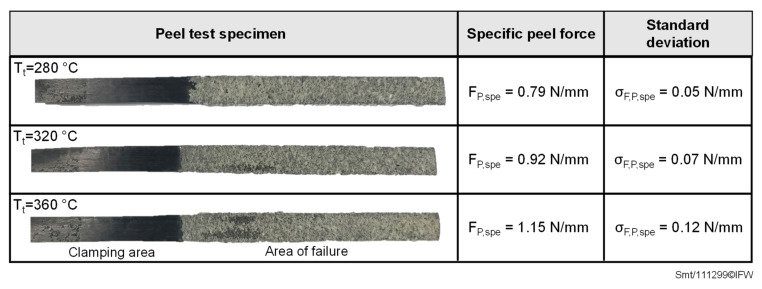
Failure patterns between deposited tape and foam structure and corresponding specific peel forces with associated standard deviations.

The specific peel forces of the peel tests, carried out to evaluate the bonding strength of the deposited prepreg tapes with the foam, are shown in Figure 8.

Increasing temperatures in the joining zone result in a higher specific peel force F_p_,_spe_. It increases from 0.79 N/mm when depositing the prepreg tape onto the foam at a temperature of 280 °C to 1.15 N/mm at a temperature of 360 °C, which corresponds to an increase of approx. 45%. For all test specimens, a thin layer of foam attached to the peeled tape surfaces can be detected (Figure 8), suggesting a cohesive failure in the foam structure. This failure pattern also occurs at temperatures below the melting temperature of the PEEK/CF prepreg tape, although a more adhesive failure pattern would have been expected here. An explanation for this finding, in combination with relatively low specific peel forces, can be found in the fact that the consolidation pressure cannot be completely applied to the heated tape and the underlying foam in the joining area, since the width of the consolidation roller (30 mm) is much wider than the width of the deposited prepreg tape (6.35 mm). During deposition, the foam surface in contact with the deposited tape is melted and deformed, but the tape cannot be completely pressed onto the melted foam layer because the deformation of the elastic roller coating is limited. The consolidation roller is instead supported on the undeformed foam areas lying to the side of the joining zone. The lack of consolidation pressure in the joining zone could lead to a weakened foam area in the boundary region between the melted foam layer attached to the tape and the underlying foam in the initial state. Grünewald [25] confirmed this phenomenon in his investigations on the production of thermoplastic sandwich structures by using the compression molding process.

### 3.2. Foam Compression Analysis

#### 3.2.1. Foam Deformation

**Figure 9:**
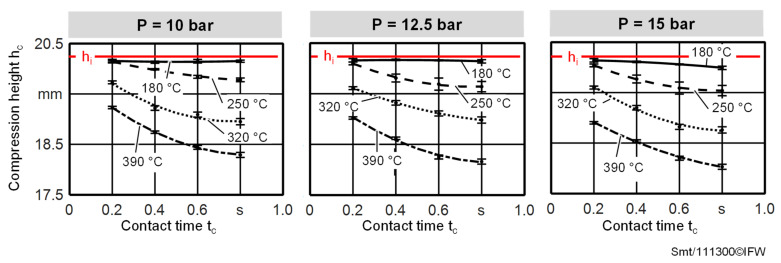
Compression heights for foam deformations for different contact times, contact temperatures and pressures.

The foam deformations of the foam compression tests as a function of the process parameters occurring in the TAFP are shown in Figure 9. Three effects can be seen here. Firstly, the foam deformation increases with increasing temperature. While for a contact temperature T_c_ of 180 °C, no significant decrease in the initial height h_i_ can be observed for the applied pressures and contact times, at temperatures above the glass transition temperature of the PEI foam (and in particular at temperatures of 320 °C and 390 °C), local melting and collapse of the foam structure occur, resulting in a significant decrease in the initial foam specimen height h_i_. Secondly, the applied pressure also has a significant influence on the resulting foam deformation. With increasing pressures, the resulting foam deformation increases. However, the selected temperature range seems to have a stronger influence on the foam deformation than the selected pressure range.

Thirdly, the foam deformation also increases with the increasing contact time, but no linear correlation between the contact time and compression height h_c_ foam deformation can be observed. Foam deformation does not increase as much with long contact times as it does with short contact times. Largest foam deformations occur at the test parameters 15 bar, 390 °C and 0.8 s for all compression tests conducted within the experimental study. The initial height is reduced by approx. 11% here. A comparison of the foam deformations determined in this isolated experimental study (with the foam deformations occurring during the deposition of tapes on the foam in the previous chapter) cannot be performed, since no roller deformations and the resulting contact pressures and contact times were determined during the fiber placement analysis in this work.

#### 3.2.2. Formation of a Melting Layer

**Figure 10:**
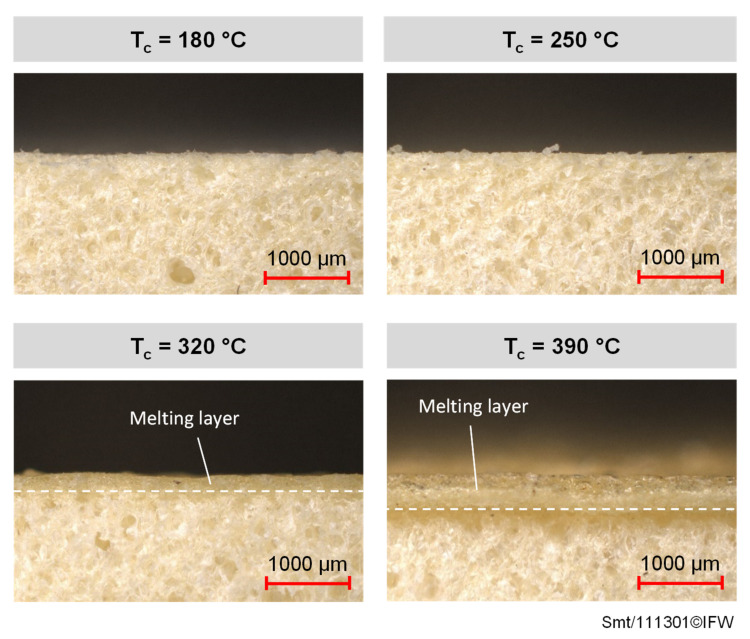
Lateral images of foam test specimen loaded under a contact time of 0.8 s, a pressure of 15 bar and different contact temperatures.

The total foam deformations of the foam specimens shown in Figure 9 are based on various effects, depending on the test parameters applied during the tests. Figure 10 shows lateral microscopic images of foam test specimens examined under the variation of temperature. The contact pressure and contact time remain constant. The foam structure in the near surface area hardly changes at contact temperatures of 180 °C and 250 °C, and the corresponding foam deformations (Figure 8) are due to a reshaping or smoothing of the rough surface in the initial state. The amorphous PEI foam material is still in a glassy, hard-elastic state. The bonds of the individual polymer chains are still strongly formed, especially at the contact temperature of 180 °C below the glass transition temperature. At the contact temperature of 320 °C, and especially at the contact temperature of 390 °C, a melting layer consisting of bulk material is formed. The polymer chains of the PEI foam material can now move more freely. The transition to a thermoplastic state causes the cell walls of the foam to deform, and the foam structure can no longer be maintained, which leads to a strong deformation in the contact area. In addition to the formation of a melting layer, it can be seen that the foam structure below the melting layer deforms slightly at the contact temperature of 390 °C due to the high thermal stress. Since this effect occurs only very locally on the outer edges of the foam test specimen, the small foam deformation area is not added to the determination of the melting layer thickness t_m_ in the next section. For all specimens shown in Figure 10, it should be noted that the internal foam structure does not change, and is therefore not affected by the applied test parameters.

**Figure 11:**
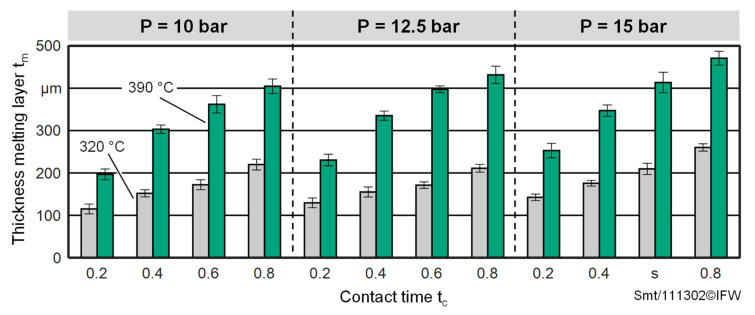
Melting layer thicknesses during loading of foam test specimens under TAFP process conditions.

Figure 11 shows the thickness of the melting layer formed by varying the test parameters. The representation is limited to the contact temperatures of 320 °C and 390 °C, since no melting layer is formed for the selected parameter space of the foam compression tests for lower temperatures. In accordance with the foam deformations investigated (Figure 9), the melting layer thickness also increases with increasing pressure, temperature and contact time. At the beginning, the melting layer is formed at the contact area between the heating plate and the foam test specimen. With increasing contact time and temperatures, a greater heat flow is transferred into the test specimen and leads to more initial foam material changing into a molten state. The melting layer subsequently continues to grow at the transition region from the melting layer and the initial foam material. The increase of the pressure could also indirectly lead to a greater heat flow into the test specimen, since both the thermal contact resistance between the heating plate and the foam test specimen and the thermal contact resistance between the melting layer and the initial foam region are reduced. It can be observed that at very low contact times, a thin melting layer and correspondingly low foam deformation already occurs. While the thinnest melting layers are in the range of approx. 110 µm, the largest melting layers are in the range of approx. 480 µm.

## 4. Discussion and Outlook

In this work, we investigated the feasibility of depositing thermoplastic prepreg tapes onto a thermoplastic foam core, for the production of purely thermoplastic sandwich structures using laser-based TAFP. The aim was to form a cohesive bond between the two joining partners and to study the effects occurring in the process. In the initial placement and in separate foam compression investigations, the following key findings were identified:

1. When PEEK/CF prepreg tape is deposited on a closed-cell PEI foam using laser-based TAFP, foam deformation occurs in the joining zone. While only moderate deformations of the foam core in the joining zone can be seen when depositing below the melting temperature of PEEK, these increase further at temperatures above the melting temperature of PEEK. Investigations of the bond strength of the deposited tapes with the foam show that peel forces increase with the increasing tape deposition temperature. A thin layer of foam attached to the peeled tape surfaces suggests a cohesive failure in the foam structure. The overall low peel forces, and the foam material attached to the peeled tape (even below the melting temperature of the PEEK/CF prepreg tape) may be the result of foam weakening in the interface between the occurring foam melting layer and the underlying undeformed foam areas, since the complete consolidation pressure could not be applied to the joining zone while the placement trials were conducted. The use of a more elastic consolidation roller could minimize this occurring effect.
2. The investigations of thermomechanical foam behavior under the TAFP process parameters showed that the applied pressure, the contact time and the contact temperature have an influence on the resulting foam deformation. An increase in these parameters leads to an increase in foam deformation. While the pressure has only a small influence on the foam deformation, the contact time and contact temperature have a greater influence on the foam deformation in the selected parameter space. In the maximum case, the initial height of the specimens is reduced from 20.2 mm to approx. 18 mm.
3. Different effects are responsible for the occurring foam deformations. At contact temperatures of 180 °C and 250 °C, a smoothing or slight reshaping of the initially rough surface leads to a decrease in the initial foam specimen height, since the amorphous PEI foam material is still in a glassy, hard-elastic state, and the bonds of the individual polymer chains are still strongly formed. At higher contact temperatures of 320 °C and 390 °C, the polymer chains of the PEI foam material can now move more freely. The transition to a thermoplastic state causes the cell walls of the foam to deform, and the foam structure can no longer be maintained. This leads to a strong deformation in the contact area and a melting layer is formed. However, the results also show that, if suitable process parameters are selected, a thin melting layer is formed for the formation of a cohesive bond, which is accompanied by only a very slight foam deformation. For all foam compression tests conducted within the experimental study, it was shown that just the very outer area of the foam test specimens was affected. This is because the foam is only exposed locally to temperature and pressure for a short time, compared to the conventional manufacturing processes of thermoplastic sandwich structures.

In the present work, it was shown that the deposition of thermoplastic tapes on a thermoplastic foam core using a laser-based TAFP is feasible. Although large deformations occurred in the joining zone when the tapes were deposited for the selected parameters, the results of the experimental study on the thermomechanical foam behavior show that, with suitable process parameters, the resulting foam deformation can be minimized and a thin melting layer required for the cohesive bonding is still formed. The present work was limited to basic investigations of the deposition of one layer of prepreg tape on a foam core. However, further effects in the production of a sandwich structure by means of a laser-based TAFP have to be examined, since the deposition of further ply layers is necessary to build up a cover layer on a foam core. In particular, two aspects that are responsible for the subsequent component quality of the sandwich structure will be investigated in future works. Firstly, the change of foam deformation and the bonding strength of the top layer to the foam core when depositing several layers of tape. Secondly, the temperature history and temperature distribution inside the deposited ply layers, as these will have a significant influence on the final cover layer quality. Furthermore, in addition to the heating strategy used in this study (sole heating of the tape), other heating strategies will be developed. The focus is on heating strategies with regard to the production of three-dimensional sandwich structures, since in this case the heat flows and the resulting temperature distributions in the joining zone change due to different geometries, and thus influence the thermomechanical interactions in the joining zone. For this purpose, the optical interactions of the two joining partners with the laser beams of the VCSEL and the resulting heat flux in the TAFP heating zone are to be investigated.

## Figures and Tables

**Table 1:** Parameter of the placement investigations.

Factor	Symbol	Unit	Value
Consolidation force	F	N	150
Tape temperature	T_T_	°C	280; 320; 360
Layup speed	v	mm/s	50

**Table 2:** Parameter set for foam compression trials.

Factor	Symbol	Unit	Value
Pressure	p	bar	10; 12.5; 15
Contact temperature	T_c_	°C	180; 250; 320; 390
Contact time	t_c_	s	0.2; 0.4; 0.6; 0.8

## Data Availability

The data presented in this study are available on request from the corresponding author.
